# Morphometric Relationship, Phylogenetic Correlation, and Character Evolution in the Species-Rich Genus *Aphis* (Hemiptera: Aphididae)

**DOI:** 10.1371/journal.pone.0011608

**Published:** 2010-07-15

**Authors:** Hyojoong Kim, Wonhoon Lee, Seunghwan Lee

**Affiliations:** 1 Division of EcoScience and Research Institute of EcoScience, Ewha Womans University, Seoul, Republic of Korea; 2 Department of Agricultural Biotechnology, Research Institute for Agriculture and Life Science, Seoul National University, Seoul, Republic of Korea; Field Museum of Natural History, United States of America

## Abstract

**Background:**

The species-rich genus *Aphis* consists of more than 500 species, many of them host-specific on a wide range of plants, yet very similar in general appearance due to convergence toward particular morphological types. Most species have been historically clustered into four main phenotypic groups (*gossypii*, *craccivora*, *fabae*, and *spiraecola* groups). To confirm the morphological hypotheses between these groups and to examine the characteristics that determine them, multivariate morphometric analyses were performed using 28 characters measured/counted from 40 species. To infer whether the morphological relationships are correlated with the genetic relationships, we compared the morphometric dataset with a phylogeny reconstructed from the combined dataset of three mtDNA and one nuclear DNA regions.

**Principal Findings:**

Based on a comparison of morphological and molecular datasets, we confirmed morphological reduction or regression in the *gossypii* group unlike in related groups. Most morphological characteristics of the *gossypii* group were less variable than for the other groups. Due to these, the *gossypii* group could be morphologically well separated from the *craccivora*, *fabae*, and *spiraecola* groups. In addition, the correlation of the rates of evolution between morphological and DNA datasets was highly significant in their diversification.

**Conclusions:**

The morphological separation between the *gossypii* group and the other species-groups are congruent with their phylogenetic relationships. Analysis of trait evolution revealed that the morphological traits found to be significant based on the morphometric analyses were confidently correlated with the phylogeny. The dominant patterns of trait evolution resulting in increased rates of short branches and temporally later evolution are likely suitable for the modality of *Aphis* speciation because they have adapted species-specifically, rapidly, and more recently on many different host plants.

## Introduction

*Aphis* Linnaeus, 1758, which contains more than 500 species, is the largest genus within the family Aphididae [1], [2]. Although attempts have been made to subdivide this species-rich genus into subgeneric groups [3], more than 80% of its species have not yet been assigned [1]. Historically, delimitation of *Aphis* species has been based on small differences in morphology, together with host associations, which have played a major part in identifying the species of this group [4], [5]. Considering that this genus contains by far the largest number of species of any genus in the family Aphididae, it is clear that *Aphis* has diversified to exploit an extremely large number of host plants [5], [6], but the morphologies of congeneric species are remarkably similar compared to those of most other genera [2], [5], [7], [8].

*Aphis* species that morphologically resemble one another have been assigned to several groups on the basis of major morphological similarities [4], [5], [9]. Stroyan [4] discussed three complexes, *Aphis fabae* Scopoli, 1763, *Aphis frangulae* Kaltenbach, 1845, and *Aphis nasturtii* Kaltenbach, 1843, each grouped by morphology and host relationships. In particular, relationships within the species-group of *A. fabae* in the European region have often been discussed based on host relationships and morphological similarities [10], [11], [12]. Some groups composed of closely-related *Aphis* species are associated with plant families, such as the *craccivora* group that feed on plants in the family Fabaceae [5]. Similarly, Pashchenko [13] assigned *Aphis* species host-specific on *Spiraea* to the *spiraecola* group.

Recently, two phylogenetic studies using molecular characters confirmed that the associations between species-groups based on morphological similarities are similar to those based on molecular characters [14], [15]. Coeur d'acier *et al.* [14] examined the phylogenetic relationships among the *craccivora* group (black-backed species), the *fabae* group (black species), the *gossypii* group (*frangulae*-like species), and the *spiraecola* group (*A. spiraecola*), and Kim & Lee [15] corroborated the phylogenetic relationships of these groups within the tribe Aphidini. These groups roughly correspond to the Latin names that have been historically applied by Börner [3] as *Cerosipha* del Guercio (*gossypii* group), *Doralis* Börner (*fabae* group), *Medoralis* Börner (*spiraecola* group), and *Pergandeida* Schouteden (*craccivora* group). Consistent with the morphology-based group designations, the molecular phylogenies showed that members within each species-group are monophyletic [14], [15]. With respect to group relationships, the *fabae* group is more closely related to the *craccivora* and *spiraecola* groups than to the *gossypii* group, whose coloration and number and length of setae differ from those of the other three groups. Although these findings imply that in *Aphis* species, genetic affinities can reflect morphological similarities [15], [16], the morphological hypotheses have not confirmed which characteristics are similar within a group or different between the groups.

Tests of the evolution morphological characters of *Aphis* species showing quite small genetic differences [14], [15] may help to better understand remarkable adaptations for wide-ranging host plants during the Tertiary [6]. Except for some polyphagous aphids such as *Aphis gossypii* Glover, 1876 and *A. fabae* utilizing more than two plant families, most *Aphis* species are confined to several host plant species within the same genus or to a few genera within the same family [2], [16]. However, *Aphis* has diversified promiscuously to various unrelated plant families unlike other aphids in the genera *Uroleucon*, *Macrosiphoniella*, and *Megoura*, which have evolved host specialization to one plant family [2], [6], [17], [18]. For example, although *A. taraxacicola*, *A. clerodendri*, *A. egomae*, and *A. sumire* within the *gossypii* group are closely related genetically and morphologically, they feed on host plants belonging to different families, namely Asteraceae, Verbenaceae, Lamiaceae, and Violaceae, respectively [15], [16]. This promiscuous host-association of *Aphis* is unusual in aphids, and their adaptation has been predicted to be related to the tendency to morphological simplicity [19].

In this study, multivariate morphometric analyses were performed for testing relationships based on morphological similarity of *Aphis*, especially comprising the four species groups, namely the *gossypii* group, the *craccivora* group, the *fabae* group, and the *spiraecola* group. In particular, morphological separations or affinities were confirmed through comparisons between the species-groups. Multivariate morphometric analysis is generally used to represent complex, multidimensional patterns of variation between host-related populations in polyphagous aphids [20], [21], [22] and has also been proven to be a powerful and reliable method for separating closely-related aphid taxa [23], [24], [25]. However, in contrast to previous studies, we employed morphometric analysis to cluster species and confirm the relationships between the previously classified species-groups in *Aphis*. Then, we evaluated the morphometric results in light of the species-group hypotheses and phylogenetic relationships proposed in previous studies [4], [5], [14], [15], and determined the major characteristics that differentiate the *Aphis* groups. In addition, we examined whether those morphological relationships are correlated with the molecular phylogeny and rates of changes [26], [27], and we tested some evolutionary features of the major characteristics using Bayesian approaches [28].

## Results

### Phylogeny

**Figure 1:**
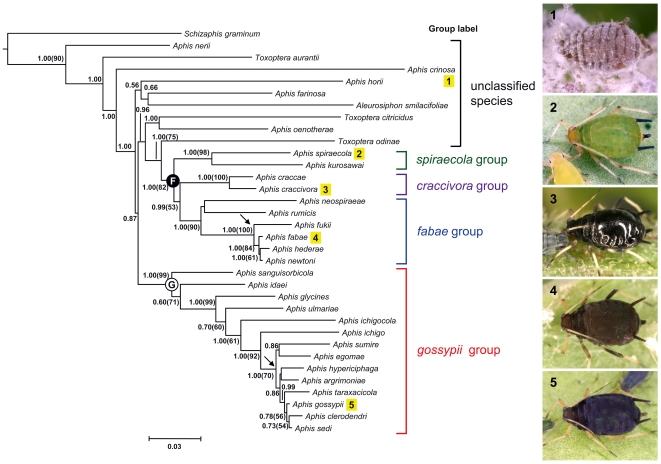
The phylogenetic relationships inferred from ML analysis based on the GTR + I + γ model using the combined sequences of COII (702 bp), 16S (1601 bp), CytB (737 bp), and EF1-α (802 bp). Posterior probabilities of BI analysis followed by ML bootstrap values in parentheses are given on or beneath nodes if greater than 50%. Letters enclosed in circles indicates two group partitions: *gossypii* group as ‘clade G’ and other three groups as ‘clade F’. Arrow means a short divergence point.

The topologies inferred by Bayesian inference (BI) and maximum likelihood (ML) analyses revealed congruent species relationships, as shown in Figure 1, although the ML bootstrap values (BL) were lower than the Bayesian posterior probabilities (PP). Although the molecular phylogeny was almost congruent to the two previous phylogenies [14], [15], we needed to combined these data to reconstruct the phylogeny for the purpose of using character reconstruction and comparison of the rate of changes (see below). Monophyly of the *gossypii* group (clade G) was highly supported (PP/BL = 1.00/99), and the *craccivora*, *spiraecola*, and *fabae* groups also formed a monophyletic group (clade F) with significant nodal support (PP/BL = 1.00/82). The three unclassified species, *A. oenotherae*, *T. citricidus*, and *T. odinae*, were closely related to clade F, and these three species and clade F formed a sister group to the *gossypii* group. The remaining unclassified species with morphological characteristics distinct from the species-groups were located in basal positions in the molecular phylogeny. Some species in the *fabae* or *gossypii* group (arrowed in Figure 1) might have undergone rapid diversification from the most recent common ancestor (MRCA) based on the small number of nucleotide substitutions differentiating species and the short branch lengths between the MRCA and the branch tips [29].

### Multivariate morphometric analysis

**Figure 2:**
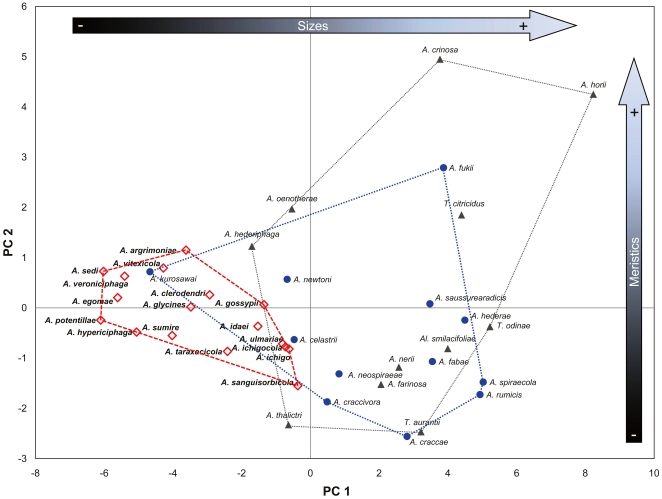
Plot of the mean scores on the first two principle components for 40 species of *Aphis* and two related genera based on 28 morphological characters. Symbols are assigned according to the preordained three groups in the text: *gossypii* group (red open diamond), *craccivora *+ *fabae *+ *spiraecola* groups (blue closed circle), and unclassified species (gray closed triangle).

In the first principal component analysis (PCA) plot based on the first two principal components (PC1, PC2), the *gossypii* group species aggregated on the mid-left side and were almost completely separate from species in the *fabae *+ *craccivora *+ *spiraecola* groups and the unclassified species, which were scattered and mixed on the right side (Table 1; Figure 2). Although some species, e.g., *A. kurosawai*, which have a small body size and short appendages, were in close proximity to species in the *gossypii* group, member species of the *gossypii* group appeared to be closely related on the basis of morphometric characters. PC1 and PC2 accounted for 50.2% and 9.4% of the total variance, respectively. Separation by PC1 was due to the length of body parts, especially the length of the antenna and its segments, the leg parts, and the body. With regard to meristic characters, the number of setae on the GP and cauda contributed substantially to the separation. Whereas, separation by PC2 was caused by certain meristic characters, such as the number of setae on Ant.I & II and the number of rhinaria on Ant.III–IV. PC3 (7.1% of the total variance) similarly contributed to the separation by both continuous and meristic characters (figure not shown). The PCA showed (Figure 2) that species in the *gossypii* group had a comparatively smaller body and shorter appendages than do species in the *fabae *+ *craccivora *+ *spiraecola* groups and unclassified species reflected by PC1, but relatively less variation in meristic characters compared to those of the other groups (PC2). In the axis of PC1, species in the *gossypii* group were distributed almost entirely according to the order of body size in length.

**Table 1:** The first two principal components that contributed to the separation of the 40 species or the 247 samples for 14 species in three PCAs.

		First PCA			Second PCA			Third PCA		
Characters		PC1	PC2	PC3	PC1	PC2	PC3	PC1	PC2	PC3
Length of	Body	0.2334	−0.1293	−0.0729	0.2517	−0.0836	0.0473	-	-	-
	Antenna	0.2503	−0.1414	0.0562	0.2545	−0.1914	−0.0160	-	-	-
	Ant.I	0.2388	−0.0809	0.0718	0.2198	−0.1059	−0.2808	-	-	-
	Ant.II	0.2377	−0.1010	−0.0629	0.2367	−0.0599	−0.1761	-	-	-
	Ant.III	0.2416	−0.1679	−0.0149	0.2497	−0.1222	0.0602	-	-	-
	Ant.IV	0.2431	−0.1692	0.0243	0.2470	−0.1878	0.0598	-	-	-
	Ant.V	0.2369	−0.1385	0.0145	0.2323	−0.2428	0.0791	-	-	-
	Ant.Vib	0.2151	−0.0989	−0.1974	0.2350	−0.1122	−0.0813	-	-	-
	PT	0.1862	−0.0694	0.3282	0.1924	−0.2339	−0.1636	-	-	-
	URS	0.1784	0.1243	0.0622	0.1999	0.1621	−0.0966	-	-	-
	HFM	0.2454	−0.1599	−0.0551	0.2544	−0.1150	0.0344	-	-	-
	HTB	0.2507	−0.1558	0.0305	0.2520	−0.1683	−0.0139	-	-	-
	2HT	0.1927	−0.0667	−0.2923	0.2276	−0.1188	−0.0814	-	-	-
	SIPH	0.1193	0.0262	0.4202	0.1067	−0.1405	0.6417	-	-	-
	Cauda	0.1287	−0.1180	0.3007	0.1325	0.1196	0.4363	-	-	-
Length of setae on	Ant.III	0.1800	0.1203	−0.3333	0.2133	0.3144	−0.1014	-	-	-
	AbdT.III	0.0847	0.1338	−0.3177	0.2002	0.2640	−0.1568	-	-	-
Number of setae on	ML	0.1766	0.1763	0.2549	0.1065	0.0696	0.3325	0.2425	−0.24516	0.0426
	Ant.I	0.1161	0.2929	0.2846	0.0768	0.2781	0.0778	0.2668	0.44432	0.3333
	Ant.II	0.1096	0.3785	0.0428	0.0293	−0.0347	0.1491	0.0484	0.76327	0.1845
	Ant.III	0.1950	0.2438	−0.2489	0.1840	0.3549	0.0456	0.4776	0.05999	−0.0050
	URS*	0.0288	0.0228	−0.1003	0.0637	0.1108	0.1963	0.1459	−0.35740	0.8538
	AbdT.VIII	0.0953	−0.0932	−0.1649	0.1514	0.3462	0.0480	0.4282	−0.03789	−0.3229
	GP	0.2152	0.0765	0.0241	0.1965	0.3141	−0.0679	0.4809	0.03411	−0.0882
	Cauda	0.2029	0.0059	−0.0036	0.2143	0.2011	−0.0149	0.4513	−0.16117	−0.1101
Number of rhinarhia on (alate)	Ant.III	0.1740	0.2508	0.0760	-	-	-	-	-	-
	Ant.IV	0.1358	0.3570	0.0171	-	-	-	-	-	-
	Ant.V	0.1080	0.4628	−0.0936	-	-	-	-	-	-
Eigenvalue		14.0478	2.6339	1.9979	13.7973	2.2484	1.4654	3.4167	1.1291	0.9789
% of total variance		50.2	9.4	7.1	55.2	9.0	5.7	42.7	14.1	12.2

Abbreviations of the characters are explained in the text.

*subsidiary setae on URS.

**Figure 3:**
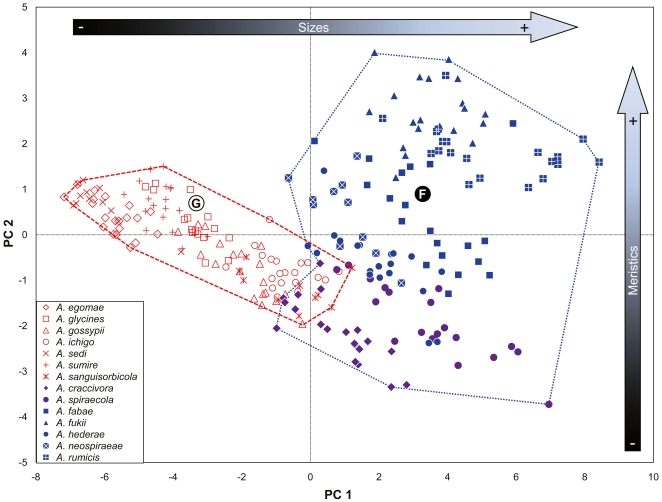
Plot of the mean scores on the first two principle components for 247 samples of 14 species representing the *gossypii* group (red-type symbols) and the *craccivora *+ *fabae *+ *spiraecola* groups (blue- and purple-type symbols) based on 25 characters.

The second PCA showed that despite highly overlapping intraspecific variation among the 14 species (Figure 3; Table 1), the *gossypii* and *fabae *+ *craccivora* groups were almost completely separated by PC1 (55.2%), PC2 (9.0%), and PC3 (5.7%), similar to the first PCA. The plots of *gossypii* group samples were concentrated on the mid-left side. In contrast, those of the *fabae *+ *craccivora* groups were largely scattered on the right side. In PC1, the separation of the axis seems to be influenced almost equally by all 14 continuous characters because there is no dominant value among them. Separation by PC2 was caused mainly by two meristic characters: the number of setae on Ant.III and the number of setae on GP. PC3 was largely affected by two continuous characters: the length of SIPH and the length of cauda (figure not shown). In the second PCA (Figure 3), the plots of the *gossypii* group samples were arranged in a narrower band on the axis of PC2 than were those of the *fabae *+ *craccivora* group samples, consistent with the first PCA. Similarly, the third PCA confirmed the high correlation of meristic characters between species in the *gossypii* group (Table 1; Figure S1). Although the plots of *A. craccivora* and *A. spiraecola* largely overlapped with that of the *gossypii* group, the plots of the former two species were clustered further than that of the *fabae* group, separated by contributions of 42.7% for PC1, 14.1% for PC2, and 12.2% for PC3. The low variation in meristic characters in the *gossypii* group revealed by the PCA analyses supported the use of these meristic characters to classify and separate the *gossypii* group from the other species-groups and the unclassified species.

The first canonical discriminate analysis (CDA) showed that CV1 (52.1% of the total variance), CV2 (17.5%), and CV3 (9.7%) separated the *gossypii* group from the *fabae *+ *craccivora* groups species (Table 2; Figure S2). The lengths of the HFM and HTB contributed significantly to the separation of both CV1 and CV2, while the separation of CV3 was dependent mainly on the length of the body and Ant.I and the number of setae on the GP and cauda. The plot patterns of the first CDA were almost identical to those of the second PCA even though samples were assigned to each species cluster in the CDA. The second CDA showed that 11 meristic characters overlapped between the *gossypii* group and *A. craccivora* (Table 2; Figure S3), but the plots of these characters in the *gossypii* group were still largely concentrated on the left side in contrast to the scattered pattern of these characters seen in the other species-groups.

**Table 2:** The first two canonical variables (total-sample standardized) that contributed to the separation of the 247 samples for 14 species in two CDAs.

		First CDA			Second CDA		
Characters		CV1	CV2	CV3	CV1	CV2	CV3
Length of	Body	0.0453	−0.0514	−1.0166	-	-	-
	Antenna	0.8638	1.3202	−0.6988	-	-	-
	Ant.I	0.6760	−0.8511	0.9101	-	-	-
	Ant.II	0.4582	−0.7713	0.6338	-	-	-
	Ant.III	−0.2235	−0.0639	0.6785	-	-	-
	Ant.IV	−0.5597	−0.0705	0.2843	-	-	-
	Ant.V	−0.6651	0.4376	−0.7908	-	-	-
	Ant.Vib	0.3510	−0.6680	0.5632	-	-	-
	PT	−0.1427	−0.9334	0.3267	-	-	-
	URS	−0.1738	0.4179	−0.1816	-	-	-
	HFM	−1.7508	1.6107	−0.4296	-	-	-
	HTB	2.0143	−2.3330	−0.6675	-	-	-
	2HT	0.6434	−1.4677	−0.8028	-	-	-
	SIPH	−0.6537	1.3879	−0.8786	-	-	-
	Cauda	−0.2051	0.1500	−0.1576	-	-	-
Length of setae on	Ant.III	1.6154	1.0154	0.6649	-	-	-
	AbdT.III	0.7460	0.4446	0.2514	-	-	-
Number of setae on	ML	0.1288	0.0017	−0.2218	0.0782	0.1852	−0.4744
	Ant.I	0.1462	0.2353	0.0127	0.2903	−0.3454	0.3121
	Ant.II	−0.0689	0.0183	0.1061	−0.0041	−0.1621	−0.0477
	Ant.III	0.4437	0.9276	0.3863	0.7454	−0.8408	−1.6160
	URS*	0.0653	0.1258	0.0415	0.0550	0.1586	−0.6803
	AbdT.VIII	0.4734	0.3713	−0.1030	0.8375	0.0696	0.3883
	GP	0.4548	−0.1828	0.8792	0.6261	−1.5202	1.1344
	Cauda	1.0266	0.3259	−1.0410	1.6037	2.2498	0.2873
Eigenvalue		29.4107	9.8950	5.4880	11.7011	2.7816	0.8378
% of total variance		52.1	17.5	9.7	71.8	17.1	5.1

*subsidiary setae on URS.

In the first ANOVA, seven continuous and two meristic characters separated the *gossypii* group from the other species groups (i.e., other all species) in the first PCA with high significance (*P*<0.0001) (Table 3). In particular, the lengths of Ant.IV and HFM among the continuous characters and the numbers of setae on the ML and cauda among the meristic characters were highly correlated with the separation of the *gossypii* group from the other species-groups. The second ANOVA showed that the 25 characters used in the second PCA that had high scores in the first PCA were also highly significant in dividing the clustered groups, except for the number of setae on Ant.II, which showed little variation. The one-way ANOVA showed that those characters were individually significant for separating the species group clusters (*gossypii* group vs. others) revealed by PCA or CDA, even although the correlated factors in multivariate analysis, e.g., size-correlation between antenna and HFM, were excluded.

**Table 3:** Statistical values by ANOVA tests for *gossypii* group versus all other species.

		First test		Second test	
Characters		*F* value	*P* value	*F* value	*P* value
Length of	Body	32.59	<.0001	410.02	<.0001
	Antenna	30.77	<.0001	307.88	<.0001
	Ant.I	20.12	0.0021	123.08	<.0001
	Ant.II	22.33	0.0001	189.34	<.0001
	Ant.III	36.36	<.0001	277.90	<.0001
	Ant.IV	40.04	<.0001	319.64	<.0001
	Ant.V	28.75	<.0001	268.54	<.0001
	Ant.VIb	21.80	0.0002	207.31	<.0001
	PT	6.03	0.0188	92.63	<.0001
	URS	8.93	0.0049	260.18	<.0001
	HFM	42.12	<.0001	410.25	<.0001
	HTB	35.49	<.0001	350.32	<.0001
	2HT	23.81	<.0001	333.32	<.0001
	SIPH	2.47	0.1242	42.34	<.0001
	Cauda	7.81	0.0081	78.69	<.0001
Length of setae on	Ant.III	16.49	0.0002	296.96	<.0001
	AbdT.III	4.41	0.0425	223.53	<.0001
Number of setae on	ML	31.98	<.0001	67.72	<.0001
	Ant.I	1.02	0.3181	10.42	<.0001
	Ant.II	1.46	0.2349	0.01	0.9085
	Ant.III	15.09	0.0004	89.65	<.0001
	URS*	2.86	0.0992	20.93	<.0001
	AbdT.VIII	2.14	0.1518	69.68	<.0001
	GP	14.17	0.0006	90.92	<.0001
	Cauda	27.15	<.0001	236.05	<.0001
Number of rhinarhia on (alate)	Ant.III	13.51	0.0007	-	-
	Ant.IV	7.79	0.0082	-	-
	Ant.V	2.68	0.1101	-	-

The first test was analyzed using 40 species, and the second test was analyzed using the 247 samples for 14 species between the groups.

*subsidiary setae on URS.

### Comparison of the morphological and molecular rates of evolution

**Figure 4:**
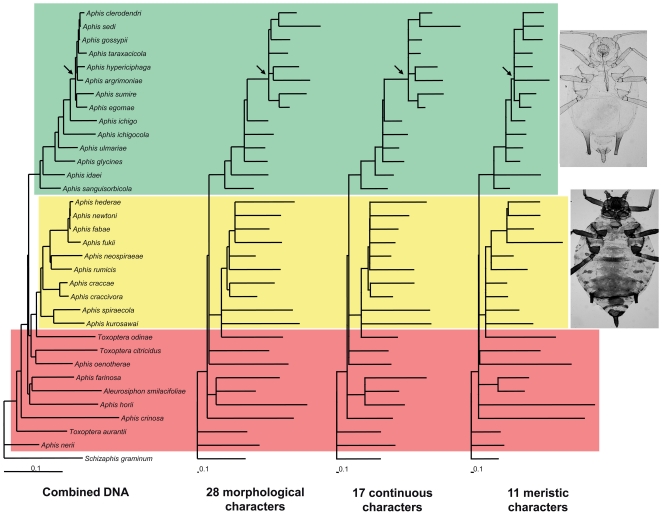
Comparison of branch lengths between the trees of combined DNA and three morphological datasets optimized with the topological constraint based on the ML tree (**Figure 1**). Arrow means a short divergence point in the *gossypii* group. *Green background* - *gossypii* group (typical morphology from *A. glycines*), *yellow* - *craccivora *+ *fabae *+ *spiraecola* groups (typical morphology from *A. craccae*), *red* - unclassified species.

Although the branch lengths of the trees generated using the Euclidean distance matrix under the topological constraint of the molecular phylogeny were different from those of the ML tree (Figures 1, 4), they were relatively similar within each classified group (clade G and clade F in Figure 4). Comparing the morphology-based trees with the DNA-based ML tree, species in the *gossypii* group had relatively shorter branches than did species in the other groups. In the two trees generated using 28 characters and 17 continuous characters, a subclade of eight species in the *gossypii* group appeared quite divergent from the remaining members, corresponding to a short branch length in the molecular phylogeny and suggestive of rapid diversification in this clade (arrowed in Figure 4). In the tree generated using the 11 meristic characters, the branch lengths of the *gossypii* group were relatively shorter than those of the other groups.

The patristic distances calculated for each morphological dataset were significantly correlated with those of the ML tree estimated from the molecular dataset based on Pearson correlation or Mantel tests (Table 4). Pearson correlation tests revealed that the molecular dataset was correlated with each morphological dataset (0.56<r<0.72; *P*<0.001). In particular, the 11 meristic characters were highly correlated with the molecular characters in Mantel test (r = 0.7378; *P* = 0.0001), while the tests with the 17 continuous character set or the 28 all character set were not significant. However, the estimated K-scores indicated that the three distance-optimized trees based on the different morphological datasets were almost congruent in topology and branch lengths, implying similar changes on nodes between the trees.

**Table 4:** Correlation of patristic distance matrices by Pearson correlation coefficient, Mantel test, and K-tree score between the molecular dataset and the different morphological dataset assessed under the ML tree topology.

	Pearson r		Mantel test		K-tree score	
Compared datasets	r	*P* value	r	*P* value	Scale factor	K-score
DNA vs morphology - 28 all characters	0.6584	<0.001	−0.0318	0.2468	0.0159	0.1965
DNA vs morphology - 17 continuous characters	0.5667	<0.001	0.0055	0.4599	0.0186	0.2376
DNA vs morphology - 11 meristic characters	0.7245	<0.001	0.7378	0.0001	0.0239	0.1956

### Analysis of phylogenetic correlation and trait evolution

The estimates of the three scaling parameters and their LR against the log-likelihood of null hypothesis' test fixing parameter (e.g., λ = 0) are listed in Table 5. The estimated values of λ were ranging from 0.50 to 0.82, of which LRs (compared with the log-likelihood when λ = 0) were estimated to be significant in chi-square distribution (*P*<0.05) except for two characters, length of cauda (*P* = 0.099) and length of setae on Ant.III (*P* = 0.052). These mean that at least 14 characters, indicating a significance of the group separation in ANOVA (Table 3), are moderately correlated with the phylogeny. The tests for the parameter κ revealed that 13 characters indicated increased rates of trait evolution in short branches (0.21<κ<0.54; *P*<0.05), but three others (length of setae on Ant.III, number of setae on ML, and number of setae on Ant.III) were rejected by null hypothesis when κ = 1 (0.88<κ<0.98; *P*>0.05), which mean that these showed a relatively gradual pace. In particular, the LRs of the 9 characters related to length were estimated to be higher than those of the other meristic characters. The estimates of the parameter δ showed that at least 6 characters indicated temporally later evolution (1.46<δ<2.47; *P*<0.05). Although the LRs were estimated relatively low (*P*>0.05), the other 8 characters ranging from 1.06 to 1.69 also suggested accelerating evolution as time progress, except for two characters, length of 2HT (δ = 0.63) and length of cauda (δ = 0.85), nominally indicating temporally early change of a trait.

**Table 5:** Evolutionary analysis of the 16 characters appeared to be significant in ANOVA, using BayesContinuous implemented in BayesTraits, showing model parameters and likelihood ratio (LR).

		Scaling parameter estimates						LR [R]andom walk vs [D]irectional
Characters		λ	LR against λ = 0	κ	LR against κ = 1	δ	LR against δ = 1	
Length of	Body	0.53	7.39**	0.30	31.31***	2.03	5.61*	2.19 [R]
	Antenna	0.67	11.66***	0.41	21.15***	1.69	3.71	<0.01 [R]
	Ant.III	0.54	9.00**	0.26	37.92***	2.10	7.35**	1.85 [R]
	Ant.IV	0.64	11.64***	0.36	22.20***	1.46	4.08*	2.52 [R]
	Ant.V	0.69	13.15***	0.33	20.98***	1.22	1.04	1.46 [R]
	HFM	0.60	15.01***	0.32	28.43***	1.64	4.04*	0.99 [R]
	HTB	0.61	13.81***	0.32	29.42***	1.69	4.61*	2.98 [R]
	2HT	0.81	14.38***	0.51	5.19**	0.63	−1.00	2.63 [R]
	Cauda	0.50	2.71	0.21	24.15***	0.85	−1.12	4.71* [R]
Length of setae on	Ant.III	0.61	3.76	0.88	3.45	2.47	4.54*	1.81† [D]
Number of setae on	ML	0.68	11.86***	0.36	17.75***	1.19	0.48	3.98* [R]
	Ant.III	0.81	12.66***	0.90	−5.81	1.89	1.92	2.82† [D]
	GP	0.82	9.24**	0.98	−0.35	2.00	2.03	0.31 [R]
	Cauda	0.72	9.73**	0.54	11.43***	1.60	3.11	0.37 [R]
Number of rhinarhia on (alate)	Ant.III	0.61	5.27*	0.37	12.88***	1.06	−4.86	0.46 [R]
	Ant.IV	0.61	4.00*	0.45	11.73***	1.13	0.15	0.13 [R]

***P<0.001.

**P<0.01.

*P<0.05.

† log-likelihood value of Model B bigger than that of Model A.

The results of LR tests for directional trends showed that the log-likelihood values of Model A (random walk) mostly tended to be larger than those of Model B (directional), but length and number of setae on Ant.III similarly showed a directional trend.

## Discussion

### Relative stasis of morphological characters in the *gossypii* group more than in other species-groups

Our morphometric analyses revealed that species in the *gossypii* group diverged more morphologically from species assigned to the *craccivora *+ *spiraecola *+ *fabae* groups [2], [4], [5]. This result, based on morphological characters, agrees well with the reciprocally monophyletic separation between the *gossypii* group and the cluster of the other three groups in the molecular phylogeny (Figure 1; [14], [15]). With respect to coloration and pigmentation, although these were not included in the morphometric analysis, the apterae of species in the *gossypii* group have a pale body color, and little or no sclerotisation or reticulation on the thoracic and abdominal dorsum [5], [16], [30].

In the morphometric analyses, species in the *gossypii* group all showed a relative reduction in the length and number of measured characters, primarily the lengths of the body, antennal segments, HFM, HTB, and the numbers of rhinaria on Ant.III and Ant.IV (Tables 1, 2; Figures 2, 3). The quantitative morphological characteristics of species in the *gossypii* group were less variable than those of species in the *craccivora *+ *spiraecola *+ *fabae* groups. The *gossypii* group generally showed reduced quantitative values for characters used in morphometric analysis, and many characters were simplified. Although the numbers of specimens for each species were insufficient to fully gauge the extent of intraspecific variation, the lengths of almost all parts of the *gossypii* group e.g., lengths of the body, antennal segments, leg parts, and setae, were relatively shorter than those of the *craccivora *+ *spiraecola *+ *fabae* groups, except for *A. kurosawai*. In addition, species in the *gossypii* group showed relatively lower within-group variation than the other species groups for most of the meristic characters used for morphometric analyses (data not shown). Consequently, relatively reductive or regressive stasis of morphological characters in the *gossypii* group made them more morphologically similar to one another than to other species-group.

### Correlation between morphological and molecular characters

Correlation between phenotypic and molecular changes is controversial [31], [32], but in *Aphis*, morphological traits appeared to be significantly correlated to molecular characters based on comparisons of rates of evolution (Figure 4; Table 4). The test of trait evolution (parameter λ in Table 5) revealed that phylogenetic signals existed in the major characters separating the *gossypii* group from the other groups, which also suggested a significant correlation between morphological and molecular evolution [28]. The sizes and numbers of characters in the *gossypii* group were significantly reduced compared to those in the other groups, and this correlation was congruent with the molecular phylogeny. Despite the fact that many species in the *gossypii* group feed on unrelated host plants, most taxonomists have assumed that these species are monophyletic and have attempted to assign species to subgeneric partitions or species-groups based on the strong affinities of morphological characters [3], [4], [5], [9]. The phylogenetic correlation between two heterogeneous characters and the correlation of their rates of evolution suggest that these characters played a significant role in the diversification of the *Aphis* group.

Furthermore, the rapid divergence of the genus *Aphis* likely affected the morphological convergence that more characterizes each species-group. The eight species in the *gossypii* group that formed a sub-cluster (arrowed in Figures 1, 4) more closely resembled one another than they did any other *Aphis* species. Similarly, *Aphis fabae*, *A. fukii*, *A. hederae*, and *A. newtoni* in the *fabae* group (arrowed in Figure 1) were also morphologically more similar to one another than to other *Aphis* species. Although we sampled only a few species in the *fabae* group, the short branch length leading to the two polyphagous species, *A. fabae* and *A. gossypii*, may be seen as evidence of a common pattern of diversification for *Aphis* species. In addition, the six subspecies [1] recognized biologically or morphologically to belong to *A. fabae* likely originated as a result of rapid diversification [33], [34]. It is worth considering that *A. fabae* and *A. gossypii* related to the rapid divergence points have both host alternation and polyphagous ability [2], [5]. For this reason, many other species morphologically similar to the *fabae* or *gossypii* group, even though they feed on the unrelated host plants, are therefore highly predicted to belong to those unique lineages.

### Evolution of the morphological characters in *Aphis*

The dominant patterns of character evolution revealed by the two parameters, κ and δ, were increased rates in short branches and temporally later evolution (Table 5; [35], [36]). These are likely suitable for the modality of *Aphis* speciation because they have species-specifically adapted for many different host plants [2], [5], [19]. The phylogenetic tree is suggestive that the *Aphis* species radiated rapidly (Figures 1, 4). For example, among the *gossypii* group, closely-related species (e.g., *A. sumire* and *A. taraxacicola*) have been become isolated in short divergence times, and this pattern of speciation also similarly appeared in the *fabae* group (arrowed in Figure 1; [14], [15]). Their relatively simple and unspecialized structures might be rather advantageous for their rapid adaptation to various hosts [19]. It is hypothesized that although the morphological adaptation seems to be less important than the physiological adaptation for survival in *Aphis* [37], [38], [39], they underwent changes in the sizes of body, legs, and antennal segments for adapting to different environments (e.g., structures of leaf, stem, and trichome) on the new host even though the change is microscopic [21]. On the other hand, it is suggested that the physiological constraint requiring the adaptation for some new host affected the variation of structures in *Aphis*, thereby their characters could be altered in a relatively short divergence time [40], [41]. It is clear that the long-term or incipient speciation pattern of *Aphis*, especially in the *gossypii* group, by the transition that they have drastically shifted their hosts to many unrelated plants is quite different from most other species in *Acyrthosiphon*, *Hyalopterus*, *Macrosiphoniella*, *Megoura*, and *Uroleucon* within Aphidinae, which have diverged to the plant species closely associated within a genus or within a family [17], [18], [42], [43]. However, similar association to unrelated hosts occurring in *Myzus persicae* [24], [44], [45] must be associated with that in *Aphis gossypii* [38], [39], [46], which seems to similarly affect the noticeable changes of these morphological characters in morphometric analysis between specialized host-adapted populations [20], [47]. These two species have a lot of similarity in regard to polyphagy, and with displaying variations in life cycle, i.e., host alternation, monoecious holocycly, or anholocycly [2], so they may have similar speciation mechanism. Due to these, if correlated between the genetic and morphological differentiation, it will be intriguing to reveal the tempo (κ, δ) of trait evolution [28] for closely-related species (e.g., *M. nicotianae*) with *M. persicae* [47]. Based on the results, it is important to investigate more whether these patterns of trait evolution appear in other aphid group, and whether these are possibly correlated with evolutionary rapid radiation.

In the analysis of trait evolution comparing Model A and Model B (Table 5), directional trends of length and number of setae on Ant.III appeared weakly even though the low LR and confidence value (*P* = 0.179 and *P* = 0.093, respectively). If a directional trend exists, species that have diverged more from the root in the phylogeny will also tend to have changed more morphologically in a given direction [28]. In fact, those characters showing directional trends contribute to identify the *Aphis* species or divide species-groups, and also have been considered as important diagnostic keys in the taxonomic studies of *Aphis* [4], [5]. Compared with that the species (e.g., *A. crinosa*, *A. horii*) in the basal position of the phylogeny have longer and much more setae on Ant.III than the others, the directional walk is likely toward regression in *Aphis*. In particular, these two characters are largely different and tend to diverge between in the *gossypii* and *fabae* groups. The former species is characterized by short and sparse setae on Ant.III, while the latter is by relatively long and dense ones. Although we cannot determine what factors historically affected these characteristics to change toward regression in this study, different convergence of these morphological characters between the species-groups need to be considered for their potential adaptive or functional ways (e.g., mechanical sensory, protective) in further study [48], [49].

## Materials and Methods

### Sampling

A total of 40 species were included in the multivariate morphometric analysis (Table S1). Only thirty-three of the above species were available from the two previous molecular phylogenies [14], [15]. Most species were classified into the species groups as suggested by previous studies [2], [4], [5], [14], [15], while the group assignments of the new species included in this study were established based on phylogenetic relationships or morphological similarities with representative species of the existing *Aphis* group. Four *Toxoptera* and *Aleurosiphon* species were also included in the study due to their close phylogenetic relationship to *Aphis* [15]. Despite the stridulatory organs that characterize *Toxoptera* [50], species in this genus are morphologically very similar to *Aphis*.

The specimens examined for this study were obtained from the Insect Museum of the College of Agriculture and Life Sciences in Seoul National University, Seoul, Rep. of Korea; the National Academy of Agricultural Science, Suwon, Rep. of Korea; and the Institute of Czech Academy of Science (Jaroslav Holman & Jan Havelka), Ceske Budejovice, Czech Republic.

### Morphometric measurements

A morphometric dataset was constructed by measuring/counting 28 characters in 40 species; 17 characters were continuous, and 11 characters were meristic (Table 1; Figure S4). To obtain the mean value of each character, we examined a total of 1,264 slide mounted specimens from 40 species (Table S2). The continuous characters measured in this study have been shown to be useful in other morphometric studies of aphids [22], [23], [25], [51], while the meristic characters were new characters adopted from morphometric and biometric analyses of *Aphis* [52], [53], [54]. Length measurements were taken using Image Lab ver. 2.2.4.0 software (MCM Design, Hillerød), and digital images were captured with a CCD, SPOT Insight™ Color Mosaic 14.2 (Diagnostic Instruments, Inc. Sterling Heights, MI) attached to a Leica DM 4000B microscope (Leica Microsystems GmbH, Wetzlar). The size and location of each character follows that described by Blackman & Eastop [2]. Morphological terminology follows that used by Heie [5] and Blackman & Eastop [2].

Abbreviations for the characters used in the text are as follows: Ant.I, Ant.II, Ant.III, Ant.IV, Ant.V, Ant.VI, Ant.VIb, antennal segments I, II, III, IV, V, VI and the base of Ant.VI, respectively; PT, processus terminalis; ML, mandibular lamina; URS, ultimate rostral segment; HFM, hind femur; HTB, hind tibia; 2HT, second segment of hind tarsus; AbdT.I, AbdT.II, AbdT.III, AbdT.IV, AbdT.V, AbdT.VI, AbdT.VII, AbdT.VIII, abdominal tergite I, II, III, IV, V, VI, VII, VIII, respectively; SIPH, siphunculus; GP, genital plate.

### Phylogeny

To reconstruct a comprehensive phylogenetic background against which to evaluate morphological relationships between the species groups and to estimate the evolution of characters, we used the phylogeny based on molecular markers which were used in the previous studies [14], [15]. Of the 40 species used in the morphological analyses, data from 33 species were available for phylogenetic reconstruction. We retrieved the sequences of two mitochondrial genes (partial tRNA-leucine + cytochrome *c* oxidase II [COII; 702 bp]; partial 12S + tRNA-valine + 16S [16S; 1601 bp]) and one nuclear gene (nuclear elongation factor 1 alpha [EF1-α; 802 bp excluding introns]) for the species including an outgroup species (*Schizaphis graminum*) from GenBank, and generated corresponding sequences for two species, *A. argrimoniae* and *A. sedi*. In addition, sequences of three species (*A. craccae*, *A. idaei*, and *A. ulmariae*) previously used by Coeur d'acier et al. [14] were also obtained from GenBank. We generated cytochrome *b* (CytB; 737 bp) sequences for 31 species in this study, as described previously [18]. For the phylogenetic analysis, the sequences of 33 ingroup species plus one outgroup were concatenated and aligned using MEGA4.0 [55] to yield a combined dataset containing ∼3,842 bp of sequence. The GenBank accession numbers of all used sequences are listed in Table S3.

A maximum likelihood (ML) tree was generated from the combined dataset. The best fit model for each gene was assessed according to the Akaike information criterion as implemented in MrModeltest 2.0 [56]. Maximum likelihood analysis was performed under a partitioned scheme for each gene, using RAxML 7.0.3 [57] with independent GTR + I + γ substitution models for each partition. Bootstrap values were estimated by RAxML based on 100 bootstrap replicates. Bayesian inference (BI) was performed using MrBayes (version 3.1.1; [58]). Markov chain Monte Carlo (MCMC) with four chains was performed for 5,000,000 generations with trees sampled every 100th generation. The best-fitting nucleotide substitution models and estimated parameters for each gene were applied separately in the MCMC analysis. The burn-in parameter was estimated empirically by plotting −ln *L* against the generation number using Tracer version 1.4 [59]; the trees corresponding to the first 10% of the generations were discarded, and the posterior probabilities were obtained by summarizing the remaining trees.

### Multivariate morphometric analysis

Principal component analysis (PCA; SAS Procedure PRINCOMP; SAS version 9.1.3; SAS Institute, Inc., Cary, NC) was carried out on the morphometric dataset of 40 species using the 28 measured/counted characters to confirm the correlations of species-groups and to determine the main components of variation in the morphological data.

Principal component analysis was performed again on the morphometric dataset of 247 apterous samples from 14 representative species; seven (*A. egomae*, *A. glycines*, *A. gossypii*, *A. ichigo*, *A. sanguisorbicola*, *A. sedi*, *A. sumire*) from the *gossypii* group and seven (*A. craccivora*, *A. fabae*, *A. fukii*, *A.hederae*, *A. neospirae*, *A. rumicis*, *A. spiraecola*) from the *fabae *+ *craccivora *+ *spiraecola* groups, using the 25 morphological characters except for three alate characters (number of rhinaria). Canonical discriminant analysis (CDA; SAS Procedure CANDISC; SAS version 9.1.3) was then performed to determine which character(s) contributed most to the separation of each species cluster. We also performed PCA and CDA using 11 meristic characters only, to exclude the effects of highly size-correlated variables between groups.

One-way analysis of variance (ANOVA; SAS Procedure ANOVA; SAS version 9.1.3) was performed for each character to evaluate the significances of differences between the *gossypii* group and all other species in the first and second PCAs.

### Comparison of the evolutionary rates between morphological and molecular characters

Using the ML tree obtained from the combined molecular dataset as a topological constraint, we compared the branch lengths estimated from the three different morphological datasets (17 continuous, 11 meristic, and 28 continuous+meristic characters) used in the first PCA. The lengths of the branches of the morphological datasets were calculated using a Euclidean distance matrix (SAS Procedure DISTANCE; SAS version 9.1.3). Comparison of the evolutionary rates between the molecular and morphological traits was conducted using PAUP*4.0b10 [60] to optimize the distance matrix and to describe the length-estimated tree on the basis of two recent studies [26], [27]. We computed patristic distances using PATRISTIC v1.0 [61] for the morphological and molecular datasets in order to test the significance of correlations by Pearson correlation coefficient (r) and Mantel test. Mantel test's significance was assessed by comparing the morphological and molecular matrices obtained in the above analysis using ZT v1.1 [62] with 1,000,000 permutations. In addition, topologies and relative branch lengths of the length-estimated trees based on each morphological dataset were compared using Ktreedist v1.0 [63].

### Hypothesis testing for the evolution of the major characters

We tested hypotheses for the evolution of the 16 major characters, which were estimated as significant according to ANOVA (Table 3), using Pagel's BayesContinuous in BayesTraits version 1.0 [28], [35]. This approach has been useful to test whether some continuous characters are correlated with the phylogeny by applying a Generalized Least Squares (GLS) method [28], [64], [65]. GLS approach provides a natural framework in which to represent some evolutionary features that arise from phylogenetic association [28]. Models of trait evolution under GLS can be compared using a likelihood-ratio (LR) test in which LR = 2[log-likelihood of the better-fitting model−log-likelihood of the worse-fitting model]. We used the harmonic mean instead of the log-likelihood as recommended by Pagel and Meade [35]. The LR statistic is asymptotically distributed as a chi-square variate with one degree of freedom [35].

We first estimated the three scaling parameters, λ (lambda), κ (kappa), and δ (delta), modeled in BayesContinuous, which can allow tests of the tempo, mode, and phylogenetic associations of trait evolution for a given phylogeny [28], [36]. The parameter λ estimates whether the phylogeny correctly predicts the patterns of covariance among species on a given trait, where λ = 0 indicates phylogenetic independence like a big-bang phylogeny, and λ = 1 denotes evolution in accordance with the topology of the phylogeny. Intermediate values of λ arise when the topology overestimates the covariance among species. The parameter κ can be used to test for a punctuational versus gradual mode of trait evolution in branches of different lengths. Values of κ<1 indicate increased rates of trait evolution in short branches, while values of κ>1 indicate that longer branches contribute more to trait evolution. A value of κ = 0 is consistent with a punctuational mode of evolution over very short time scales. The parameter δ estimates whether the rate of trait evolution has accelerated or slowed over time as one moves from the root to the tips of the tree. Values of δ<1 can indicate temporally early evolution of a trait (i.e., adaptive radiation), while values of δ>1 indicate temporally later evolution of a trait (i.e., species-specific adaptation). Values of κ = 1 or δ = 1 are interpreted as a default gradualism in each case. For determining the confidence of each estimated parameter, the LR were obtained from tests for the estimated value against little phylogenetic correlation (λ = 0), or a gradual pace (δ = 1, κ = 1). Secondly, we estimated directional trends by comparing Model A (random walk) with Model B (directional walk) via their log-likelihoods [28], [35]. The random walk of Model A is sometimes called as a Brownian motion. If Model B fits the data better (larger log-likelihood – closer to zero) than Model A, it says that a directional trend exists.

For each test, log-likelihood and rate parameter values were explored to find acceptance rates when running the Markov chains of between 20 and 40% as recommended by Pagel and Meade [35], [66]. The Markov chain was run for 10 million generations sampled every 1000th generations after a burnin of a million generation. Convergence of the model was investigated by finding a stationary phase of the harmonic means.

## Supporting Information

Figure S1 Plot of the mean scores on the first two principle components for 14 species representing the gossypii group (red-type symbols) and the craccivora + fabae + spiraecola groups (blue- and purple-type symbols) based on 11 meristic characters.
(3.15 MB TIF)

Figure S2 Plot of the mean scores on the (A) CV1 vs CV2 and (B) CV1 vs CV3 for 14 species representing the gossypii (open circle), the craccivora (blue closed triangle), the spiraecola (red closed square), and the fabae (black closed diamond) groups based on 25 characters.
(2.35 MB TIF)

Figure S3 Plot of the mean scores on the (A) CV1 vs CV2 and (B) CV1 vs CV3 for 14 species representing the gossypii (open circle), the craccivora (blue closed triangle), the spiraecola (red closed square), and the fabae (black closed diamond) groups based on 11 meristic characters.
(2.25 MB TIF)

Figure S4 Picture of the structures and reference parts employed in the morphometric analyses (represented by A. craccivora; aptera (A–G) and alata (H)): A, body; B, antennal segment III; C, second hind tarsal segment; D, ultimate rostral segment; E, seta on abdominal tergite III; F, cauda; G, genital plate; H, antennal segment III–V. Abbreviations are explained in the text.
(5.16 MB TIF)

Table S1 Taxonomic, biological, and species-group information of species used for morphometric analysis.
(0.10 MB DOC)

Table S2 Samples of apterous (Ap.) and alate (Al.) viviparae of species measured/counted for morphometric analysis.
(0.07 MB DOC)

Table S3 GenBank accession numbers of the sequences used in this study.
(0.07 MB DOC)
