# Supplementary figures and images for: Morphometric Relationship, Phylogenetic Correlation, and Character Evolution in the Species-Rich Genus Aphis (Hemiptera: Aphididae)
Source: PLoS One. 2010 Jul 15;5(7):e11608. doi: 10.1371/journal.pone.0011608 (PMC2904707; doi:10.1371/journal.pone.0011608)

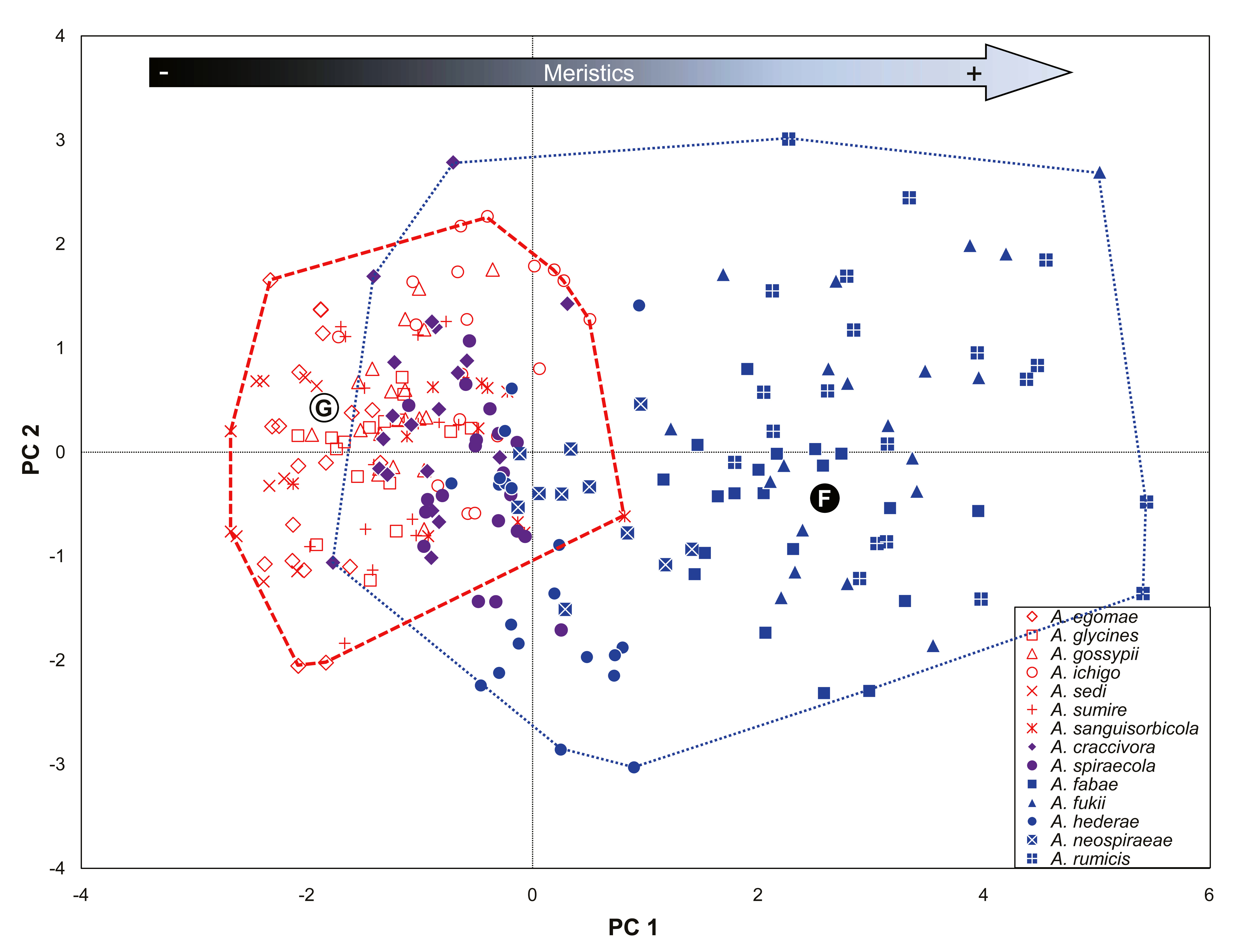

Supplement: Figure S1 — Plot of the mean scores on the first two principle components for 14 species representing the gossypii group (red-type symbols) and the craccivora + fabae + spiraecola groups (blue- and purple-type symbols) based on 11 meristic characters. (3.15 MB TIF) [file pone.0011608.s001.tif]

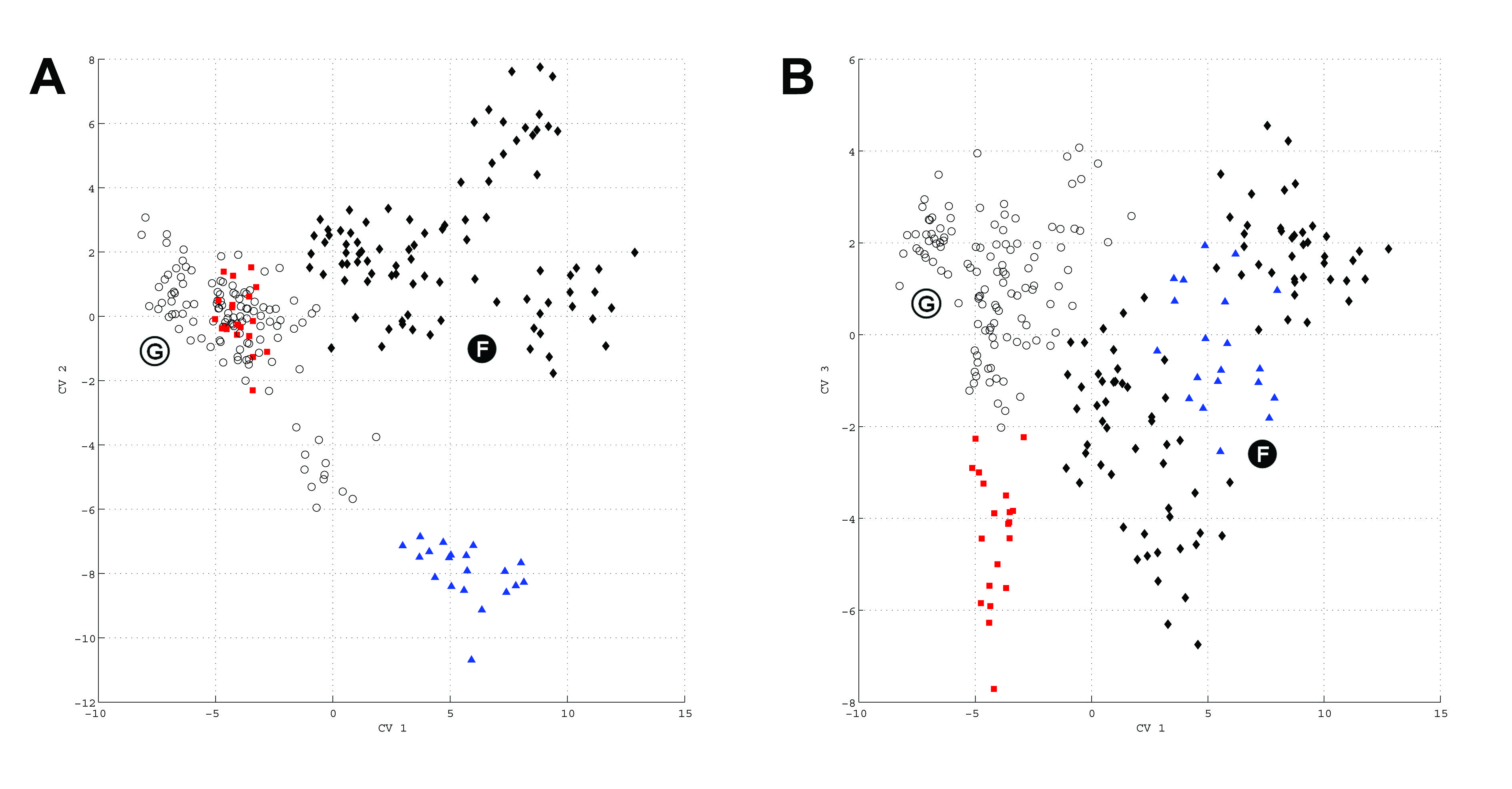

Supplement: Figure S2 — Plot of the mean scores on the (A) CV1 vs CV2 and (B) CV1 vs CV3 for 14 species representing the gossypii (open circle), the craccivora (blue closed triangle), the spiraecola (red closed square), and the fabae (black closed diamond) groups based on 25 characters. (2.35 MB TIF) [file pone.0011608.s002.tif]

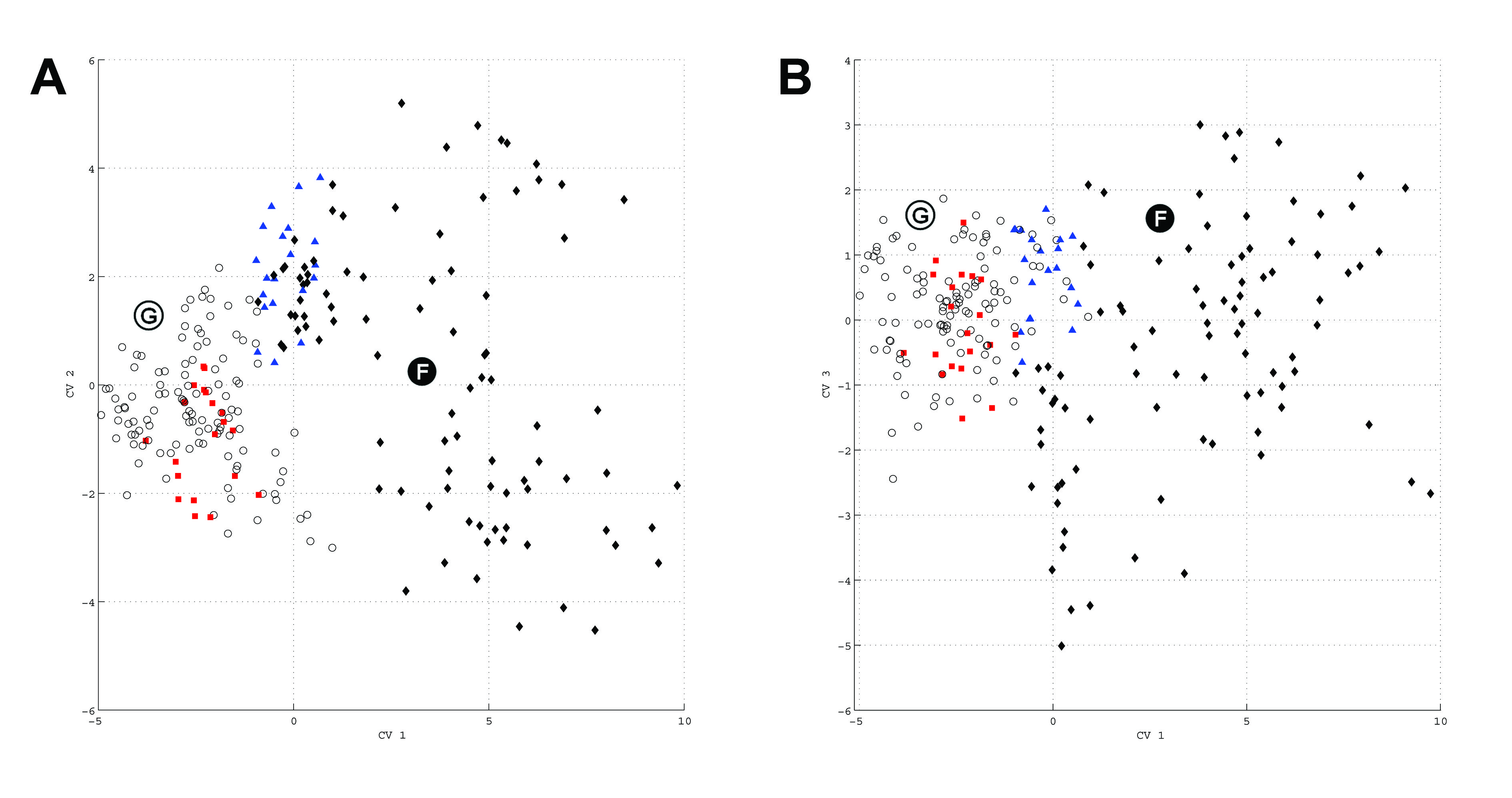

Supplement: Figure S3 — Plot of the mean scores on the (A) CV1 vs CV2 and (B) CV1 vs CV3 for 14 species representing the gossypii (open circle), the craccivora (blue closed triangle), the spiraecola (red closed square), and the fabae (black closed diamond) groups based on 11 meristic characters. (2.25 MB TIF) [file pone.0011608.s003.tif]

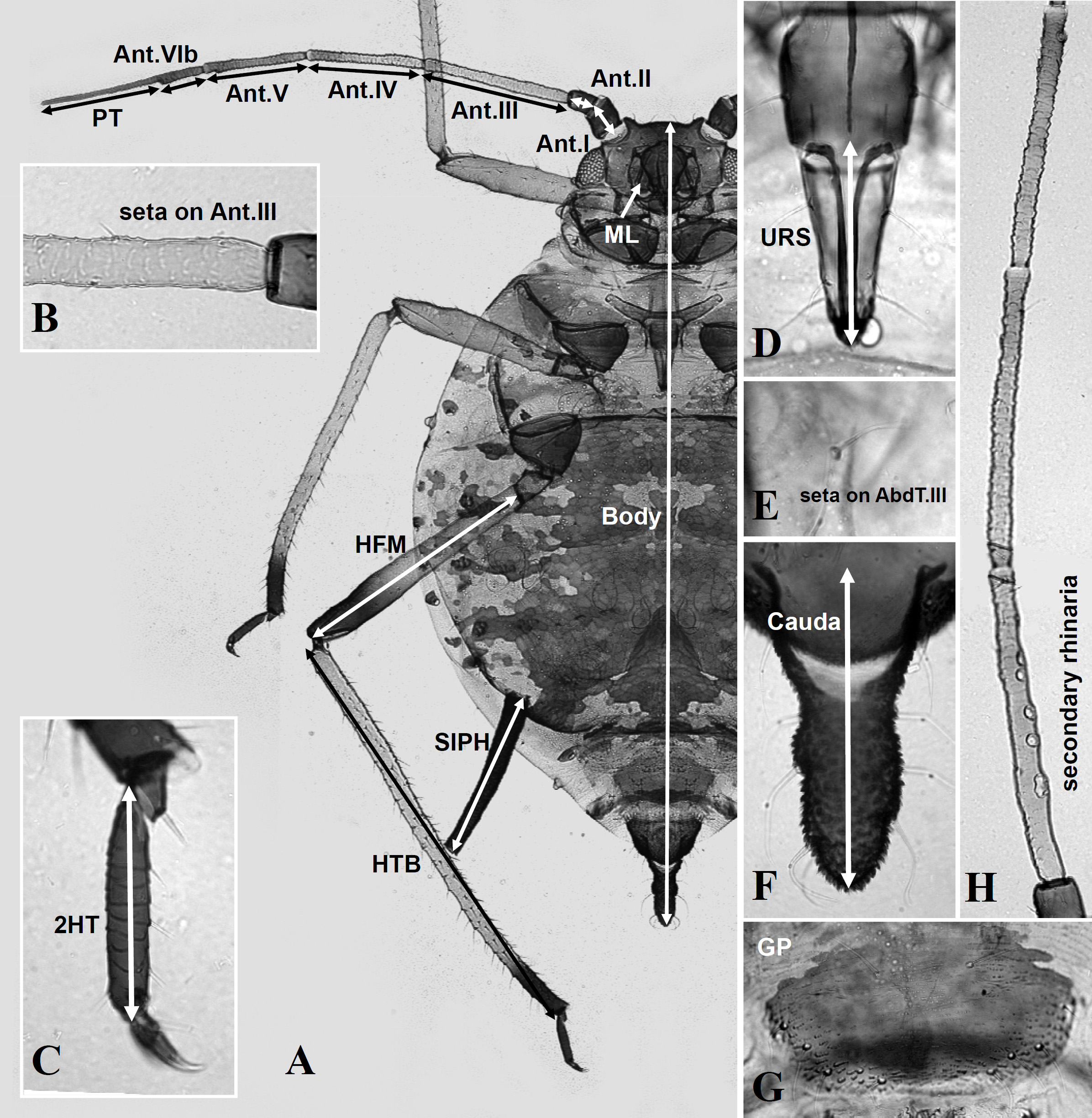

Supplement: Figure S4 — Picture of the structures and reference parts employed in the morphometric analyses (represented by A. craccivora; aptera (A–G) and alata (H)): A, body; B, antennal segment III; C, second hind tarsal segment; D, ultimate rostral segment; E, seta on abdominal tergite III; F, cauda; G, genital plate; H, antennal segment III–V. Abbreviations are explained in the text. (5.16 MB TIF) [file pone.0011608.s004.tif]
