# Supplementary material for: Morphometric Relationship, Phylogenetic Correlation, and Character Evolution in the Species-Rich Genus Aphis (Hemiptera: Aphididae)
Source: PLoS One. 2010 Jul 15;5(7):e11608. doi: 10.1371/journal.pone.0011608 (PMC2904707; doi:10.1371/journal.pone.0011608)
Supplement: Table S1 — Taxonomic, biological, and species-group information of species used for morphometric analysis. (0.10 MB DOC) [file pone.0011608.s005.doc]

| **Species*** | **Trophic specificity** | **Life cycle†** | **Distribution** | **Species-group†** | **Reference‡** |
| --- | --- | --- | --- | --- | --- |
| *Aphis* (*Aphis*) *argrimoniae* (Shinji, 1941) | Monophagous | Mon. ? | EA | *gossypii* group ? | - |
| *Aphis* (*Aphis*) *celastrii* Matsumura, 1917 | Oligophagous | Mon. / Heter. | EA | *spiraecola* group ? | - |
| *Aphis* (*Aphis*) *clerodendri* Matsumura, 1917 | Monophagous | Mon. ? | AU, EA | *gossypii* group | B, K |
| *Aphis* (*Aphis*) *craccae* Linnaeus, 1758 | Oligophagous | Mon. | NA, PA | *craccivora* group | B, C, H, S, K |
| *Aphis* (*Aphis*) *craccivora* Koch, 1854 | Polyphagous | Mon. / Anhol. | Cosmopolitan | *craccivora* group | C, H, S, K |
| *Aphis* (*Aphis*) *crinosa* Paik, 1969 | Oligophagous | Mon. ? | EA | unclassified | - |
| *Aphis* (*Aphis*) *egomae* Shinji, 1922 | Monophagous | Mon. ? | EA | *gossypii* group | K |
| *Aphis* (*Aphis*) *fabae* Scopoli, 1763 | Polyphagous | Heter. | Cosmopolitan | *fabae* group | H, S, K |
| *Aphis* (*Aphis*) *farinosa* J.F. Gmelin, 1790 | Monophagous | Mon. ? | NA, IM, PA | unclassified | - |
| *Aphis* (*Aphis*) *fukii* Shinji, 1922 | Monophagous | Mon. ? | EA | *fabae* group | K |
| *Aphis* (*Aphis*) *glycines* Matsumura, 1917 | Monophagous | Heter. | EA, NA, IM | *gossypii* group | K |
| *Aphis* (*Aphis*) *gossypii* Glover, 1877 | Polyphagous | Mon. / Anhol. | Cosmopolitan | *gossypii* group | B, C, H, S, K |
| *Aphis* (*Aphis*) *hederae* Kaltenbach, 1843 | Oligophagous | Mon. | NA, PA | *fabae* group | S, K |
| *Aphis* (*Aphis*) *hederiphaga* Pashtshenko, 1933 | Monophagous | Mon. ? | EA | unclassified | - |
| *Aphis* (*Aphis*) *horii* Takahashi, 1923 | Oligophagous | Heter. | PA | unclassified | - |
| *Aphis* (*Aphis*) *hyperciphaga* Pashtshenko, 1933 | Monophagous | Mon. ? | EA | *gossypii* group | K |
| *Aphis* (*Aphis*) *ichigo* Shinji, 1922 | Oligophagous | Mon. ? | EA | *gossypii* group | K |
| *Aphis* (*Aphis*) *ichigocola* Shinji, 1924 | Oligophagous | Mon. ? | EA, IM | *gossypii* group | K |
| *Aphis* (*Aphis*) *idaei* van der Goot, 1912 | Monophagous | Mon. | AU, PA | *gossypii* group ? | - |
| *Aphis* (*Aphis*) *kurosawai* Takahashi, 1921 | Monophagous | Mon. ? | EA, IM | *spiraecola* group | K |
| *Aphis* (*Aphis*) *neospiraeae* Takahashi, 1966 | Monophagous | Mon. | EA, IM | *fabae* group | K |
| *Aphis* (*Aphis*) *nerii* Boyer de Foscolombe, 1841 | Monophagous | Mon. | Cosmopolitan | unclassified | - |
| *Aphis* (*Aphis*) *newtoni* Theobald, 1927 | Oligophagous | Mon. | EA, EU | *fabae* group | C, H, S, K |
| *Aphis* (*Aphis*) *potentillae* Nevsky, 1929 | Monophagous | Mon. ? | EA | *gossypii* group ? | - |
| *Aphis* (*Aphis*) *rumicis* Linnaeus, 1758 | Oligophagous | Mon. | NA, IM, PA | *fabae* group | S, C, K |
| *Aphis* (*Aphis*) *sanguisorbicola* Takahashi, 1966 | Monophagous | Mon. | EA | *gossypii* group | K |
| *Aphis* (*Aphis*) *saussurearadicis* Pashtshenko, 1992 | Monophagous | Mon. | EA | *fabae* group ? | - |
| *Aphis* (*Aphis*) *sedi* Kaltenbach, 1843 | Monophagous | Mon. | AU, NA, PA | *gossypii* group | H, S, K |
| *Aphis* (*Aphis*) *spiraecola* Patch, 1914 | Polyphagous | Heter. / Anhol. | Cosmopolitan | *spiraecola* group | K |
| *Aphis* (*Aphis*) *sumire* Moritsu, 1949 | Monophagous | Mon. ? | EA | *gossypii* group | K |
| *Aphis* (*Aphis*) *taraxacicola* (Borner, 1940) | Monophagous | Mon. | PA | *gossypii* group | K |
| *Aphis* (*Aphis*) *thalictri* Koch, 1854 | Monophagous | Mon. | PA | unclassified | C |
| *Aphis* (*Aphis*) *ulmariae* Schrank, 1801 | Monophagous | Mon. | PA, NA | *gossypii* group ? | C |
| *Aphis* (*Aphis*) *veroniciphaga* Kim et Lee, 2006 | Monophagous | Mon. ? | EA | *gossypii* group ? | - |
| *Aphis* (*Aphis*) *vitexicola* Kim et Lee, 2006 | Monophagous | Mon. ? | EA | *gossypii* group ? | - |
| *Aphis* (*Bursaphis*) *oenotherae* Oestlund, 1887 | Oligophagous | Heter. / Anhol. | EA, EU, NA | unclassified | C, K |
| *Aleurosiphon smilacifoliae* (Takahashi, 1921) | Monophagous | Mon. | EA | unclassified | K |
| *Toxoptera aurantii* (Boyer de Fonscolombe, 1841) | Polyphagous | Anhol. | Cosmopolitan | unclassified | K |
| *Toxoptera citricidus* (Kirkaldy, 1907) | Polyphagous | Anhol. | AU, EA, IM, NA | unclassified | K |
| *Toxoptera odinae* (van der Goot, 1917) | Polyphagous | Anhol. | EA, IM | unclassified | K |

Abbreviations: *Life cycle* - Anhol., anholocycly; Heter., heteroecious holocycly, Mon., monoecious holocycly; *Distribution* - AF, Afrotropic; AU, Australasia; EA, Eastern Asia; EU, Europe; IM, Indomalaya; NA, Nearctic; NE, Neotrophic; PA, Palearctic; *Reference* - B, Blackman and Eastop [2]; C, Coeur d’acier et al. [14]; H, Heie [5]; K, Kim and Lee [15]; S, Stroyan [4]

*Scientific name and classification following Remaudiére and Remaudiére [1] except *T. citricidus* following Nieto Nafría et al. [67].

† Question mark hypothesized or classified in this study

‡ Proposed from previous studies
