# Supplementary material for: Morphometric Relationship, Phylogenetic Correlation, and Character Evolution in the Species-Rich Genus Aphis (Hemiptera: Aphididae)
Source: PLoS One. 2010 Jul 15;5(7):e11608. doi: 10.1371/journal.pone.0011608 (PMC2904707; doi:10.1371/journal.pone.0011608)
Supplement: Table S2 — Samples of apterous (Ap.) and alate (Al.) viviparae of species measured/counted for morphometric analysis. (0.07 MB DOC) [file pone.0011608.s006.doc]

| Species | No. of specimens (Ap./Al.) | Host plant [Family] | Locality * | Date † |
| --- | --- | --- | --- | --- |
| *Aphis argrimoniae* | 10/10 | *Agrimonia pilosa* [Rosaceae] | SK: Hamyang | 21.IV.99 |
| *Aphis celastrii* | 10/10 | *Celastrus orbiculatus* [Celastraceae] | SK: Suwon | 5.VI.03 |
| *Aphis clerodendri* | 10/10 | *Clerodendrum trichotomum* [Lamiaceae] | SK: Boreyong | 9.X.03 |
| *Aphis craccae* | 10/10 | *Vicia* (*cracca*, *venosa*) [Fabaceae] | NK: Hyesan, Mupo | 8.VII.85, 14.VII.87 |
| *Aphis craccivora* | 20/20 | *Capsella bursa-pastoris* [Brassicaceae], *Glycine soja*, *Glycyrrhiza uralensis*, *Trifolium repens*, *Phaseolus radiatus* [Fabaceae] | SK: Gurye, Suwon, Chengyang, Hamyang | 4.V.99, 15.VI.99, 6.VII.99, 20.VI.00 |
| *Aphis crinosa* | 20/19 | *Ligustrum* *obtusifolium* [Oleaceae] | NK: Soktam, SK: Jinan | 31.V.87, 13.V.99 |
| *Aphis egomae* | 20/20 | *Perilla frutescens* [Lamiaceae] | SK: Suwon, Ulreung, Heongseong | 24.VII.98, 8.VI.00, 5.VIII.05 |
| *Aphis fabae* | 20/20 | *Hemistepta lyrata*, *Youngia japonica* [Asteraceae ], *Rumex crispus*, *Reynoutria sachalinensis* [Polygonaceae], *Solanum nigrum* [Solanaceae], *Anthriscus sylvestris* [Apiaceae] | SK: Samcheok, Jeju, Namhae, Muan, Urleung, Namjeju, Busan | 23.V.98, 11.VI.98, 30.III.99, 1.VI.99, 5.VI.00, 26.X.00, 13.V.04 |
| *Aphis farinosa* | 20/20 | *Salix* sp. [Salicaceae] | SK: Samcheok, Anyang | 23.V.98, 3.VI.05 |
| *Aphis fukii* | 20/20 | *Petasites japonicus* [Asteraceae] | SK: Bukjeju, Boreyong, Pyeongchang | 24.X.00, 9.X.03, 18.VII.04 |
| *Aphis glycines* | 16/17 | *Glycine* (*max*, *soja*) [Fabaceae] | SK: Gimhae, Danyang, Pyeongchang | 17.IX.98, 22.X.99, 17.IX.04 |
| *Aphis gossypii* | 20/20 | *Althaea rosea*, *Gossypium indicum*, *Hibiscus* *mutabilis* [Malvaceae], *Leonurus sibiricus* [Lamiaceae], *Celastrus orbiculatus*, *Cucumis sativus* [Cucurbitaceae], *Chrysanthemum morifolium*, *Ixeris dentata* [Asteraceae] | SK: Yesan, Pyeongchang, Pohang, Jeju, Naju, Gimcheon, Seogwipo | 22.IV.98, 20.V.98, 28.V.98, 19.VI.98, 12.V.99, 14.V.99, 27.V.04 |
| *Aphis hederae* | 20/20 | *Hedera* *rhombea*, *Schefflera actinophylla* [Araliaceae] | SK: Jeju, Bukjeju | 25.X.00, 14.V.03 |
| *Aphis hederiphaga* | 20/10 | *Hedera* *rhombea* [Araliaceae] | SK: Bukjeju, Namjeju | 24.X.00, 27.V.04 |
| *Aphis horii* | 20/20 | *Sambucus* sp. [Caprifoliaceae] | SK: Jeju, Suwon, Ulreung | 24.V.00, 29.V.01, 8.VI.04 |
| *Aphis hyperciphaga* | 14/19 | *Hypericum ascyron* [Clusiaceae] | NK: Pyeongyang, SK: Inje, Yangyang | 2.VI.88, 4.VI.99, 17.VI.05 |
| *Aphis ichigo* | 20/18 | *Rubus* (*coreanus*, *crataegifolius*) [Rosaceae] | NK: Kumgangsan SK: Ulreung, Seoul | 26.V.88, 6.VI.00, 25.V.04 |
| *Aphis ichigocola* | 12/16 | *Rubus* (*crataegifolius, parvifolius*) [Rosaceae] | NK: Pyeongyang, SK: Jeju | 8.VI.85, 14.V.03 |
| *Aphis idaei* | 12/10 | *Rubus phoenicolasius* [Rosaceae] | NK: Paektusan | 26.VI.88 |
| *Aphis kurosawai* | 18/20 | *Artemisia* *princeps* [Asteraceae] | SK: Sobaeksan, Jeju, Gwangyang | 21.V.99, 25.X.00, 21.VI.04 |
| *Aphis neospiraeae* | 11/18 | *Spiraea* (*japonica*, *prunifolia*) [Rosaceae] | SK: Pocheon, Chuncheon, Suwon | 21.V.99, 10.VII.00, 23.V.03 |
| *Aphis nerii* | 20/20 | *Asclepias* sp., *Nerium indicum*, *Pleuropterus multiflorus* [Apocynaceae] | SK: Yeongwol, Hoengseong, Bukjeju | 10.IX.98, 11.VIII.99, 18.VI.00 |
| *Aphis newtoni* | 17/17 | *Iris* *sanguinea* [Iridaceae] | SK: Suwon, Paju, Anyang | 15.X.99, 14.V.01, 3.VI.05 |
| *Aphis potentillae* | 10/14 | *Potentilla dickinsii* [Rosaceae] | NK: Kumgangsan, Pyeongyang, SK: Pyeongchang | 4.VI.87, 11.VI.88, 25.VI.03 |
| *Aphis rumicis* | 20/20 | *Rumex crispus* [Polygonaceae] | SK: Pyeongchang, Bukjeju, Ulreung | 8.VI.98, 17.VI.00, 8.VI.04 |
| *Aphis sanguisorbicola* | 10/12 | *Sanguisorba officinalis* [Rosaceae] | SK: Suwon, Chuncheon | 14.VII.88, 16.IV.93 |
| *Aphis saussurearadicis* | 17/14 | *Ligularia fischeri*, *Saussurea pulchella* [Asteraceae] | NK: Mupo, Hyesan | 11.VII.85, 11.VII.87 |
| *Aphis sedi* | 12/10 | *Sedum kamtschaticum* [Crassulaceae] | SK: Jinan, Seoul | 13.V.99, 11.V.03 |
| *Aphis spiraecola* | 20/20 | *Malus pumila*, *Pyrus ussuriensis* [Rosaceae], *Gerbera jamsonii*, *Chrysanthmum morifolium* [Asteraceae], *Citrus* (*junos*, *unshiu*) [Rutaceae], *Pittosporum tobira* [Pittosporaceae] | SK: Chungju, Gunwi, Pyeongchang, Jeju, Namhae, Jeju | 3.VI.97, 15.VII.97, 15.X.97, 19.VI.98, 30.VI.98, 25.X.00 |
| *Aphis sumire* | 18/14 | *Viola dissecta* [Violaceae] | SK: Boreyong, Wonju, Mungyeong | 9.XI.03, 9.XI.04, 20.V05 |
| *Aphis taraxacicola* | 11/12 | *Taraxacum officinale* [Asteraceae] | SK: Suwon, Mungyeong | 13.V.05, 20.V.05 |
| *Aphis thalictri* | 14/11 | *Thalictrum* (*contortum*, *simplex*) [Ranunculaceae] | NK: Paektusan, Samjiyon | 10.VI.85, 21.VI.88 |
| *Aphis ulmariae* | 13/13 | *Filipendula palmata* [Rosaceae] | NK: Paektusan, Yongkwang | 21.VI.88, 29.VI.88 |
| *Aphis veroniciphaga* | 17/10 | *Veronica nakaiana* [Scrophulariaceae] | SK: Ulreung | 8.VI.00 |
| *Aphis vitexicola* | 15/11 | *Vitex nedundo* [Lamiaceae] | SK: Jumunjin | 3.VI.99 |
| *Aphis* (*Bursaphis*) *oenotherae* | 20/20 | *Oenothera odorata* [Onagraceae] | SK: Inje, Hoengseong, Busan | 4.VI.99, 25.VI.03, 13.V.04 |
| *Aleurosiphon smilacifoliae* | 20/20 | *Smilax china* [Smilacaceae] | SK: Tongyeong, Bukjeju, Busan | 13.V.03, 14.V.04, 11.V.06 |
| *Toxoptera aurantii* | 16/12 | *Camellia japonica* [Theaceae] | SK: Suwon, Yeosu, Namjeju | 7.IV.98, 7.X.99, 20.IV.00 |
| *Toxoptera citricidus* | 17/11 | *Cudrania tricuspidata* [Moraceae] | SK: Gwangyang, Jeju | 21.VI.04, 27.V.04 |
| *Toxoptera odinae* | 12/14 | *Rhus* (*chinensis*, *verniciflua*) [Anacardiaceae] | SK: Jeju, Jinan | 12.VI.98, 20.VI.00 |

* SK, South Korea; NK, North Korea. † Same order of locality.
