# Supplementary material for: Morphometric Relationship, Phylogenetic Correlation, and Character Evolution in the Species-Rich Genus Aphis (Hemiptera: Aphididae)
Source: PLoS One. 2010 Jul 15;5(7):e11608. doi: 10.1371/journal.pone.0011608 (PMC2904707; doi:10.1371/journal.pone.0011608)
Supplement: Table S3 — GenBank accession numbers of the sequences used in this study. (0.07 MB DOC) [file pone.0011608.s007.doc]

| no. | scientific name | specimen voucher | COII | CytB | 16S | EF1α |
| --- | --- | --- | --- | --- | --- | --- |
| 1 | *Aphis argrimoniae* | 080925HJ29 | GQ904149 | GU205342 | GU205373 | GU205375 |
| 2 | *Aphis clerodendri* | 031009SH32 | EU358824 | GU205343 | EU358864 | EU358904 |
| 3 | *Aphis craccae* | Coeur d’acier et al. [14] | AM085421 | AM085376 | - | - |
| 4 | *Aphis craccivora* | 031026HJ1 | EU358825 | GU205344 | EU358865 | EU358905 |
| 5 | *Aphis crinosa* | 050425SH1 | EU358826 | GU205345 | EU358866 | EU358906 |
| 6 | *Aphis egomae* | 050809HJ1 | EU358827 | GU205346 | EU358867 | EU358907 |
| 7 | *Aphis fabae* | 040527HJ16 | EU358828 | GU205347 | EU358868 | EU358908 |
| 8 | *Aphis farinosa* | 030523SH14 | EU358829 | GU205348 | EU358869 | EU358909 |
| 9 | *Aphis fukii* | 040527HJ10 | EU358830 | GU205349 | EU358870 | EU358910 |
| 10 | *Aphis glycines* | 040917HJ3 | EU358831 | GU205350 | EU358871 | EU358911 |
| 11 | *Aphis gossypii* | 030513HJ47 | EU358832 | GU205351 | EU358872 | EU358912 |
| 12 | *Aphis hederae* | 060407SH24 | EU358833 | GU205352 | EU358873 | EU358913 |
| 13 | *Aphis horii* | 040608HJ24 | EU358834 | GU205353 | EU358874 | EU358914 |
| 14 | *Aphis hypericiphaga* | 050616SH29 | EU358835 | GU205354 | EU358875 | EU358915 |
| 15 | *Aphis ichigo* | 030625SH53 | EU358836 | GU205355 | EU358876 | EU358916 |
| 16 | *Aphis ichigocola* | 030513SH10 | EU358837 | GU205356 | EU358877 | EU358917 |
| 17 | *Aphis idaei* | Coeur d’acier et al. [14] | AM085413 | AM085368 | - | - |
| 18 | *Aphis kurosawai* | 050603HJ16 | EU358838 | GU205357 | EU358878 | EU358918 |
| 19 | *Aphis neospiraeae* | 030523SH25 | EU358839 | GU205358 | EU358879 | EU358919 |
| 20 | *Aphis nerii* | 031009SH25 | EU358840 | GU205359 | EU358880 | EU358920 |
| 21 | *Aphis newtoni* | 050603SH10 | EU358841 | GU205360 | EU358881 | EU358921 |
| 22 | *Aphis rumicis* | 040513HJ10 | EU358843 | GU205361 | EU358883 | EU358923 |
| 23 | *Aphis sanguisorbicola* | 040917HJ6 | EU358844 | GU205362 | EU358884 | EU358924 |
| 24 | *Aphis sedi* | 030511HJ1 | GQ904161 | GU205363 | GU205374 | GU205376 |
| 25 | *Aphis spiraecola* | 050603HJ6 | EU358845 | GU205364 | EU358885 | EU358925 |
| 26 | *Aphis sumire* | 050513HJ21 | EU358846 | GU205365 | EU358886 | EU358926 |
| 27 | *Aphis taraxacicola* | 050513HJ11 | EU358847 | GU205366 | EU358887 | EU358927 |
| 28 | *Aphis ulmarie* | Coeur d’acier et al. [14] | AM085414 | AM085369 | - | - |
| 29 | *Aphis oenotherae* | 030625SH67 | EU358842 | GU205367 | EU358882 | EU358922 |
| 30 | *Aleurosiphon smilacifoliae* | 040513HJ22 | EU358823 | GU205368 | EU358863 | EU358903 |
| 31 | *Toxoptera aurantii* | 030513SH107 | EU358860 | GU205369 | EU358900 | EU358940 |
| 32 | *Toxoptera citricidus* | 040621SH20 | EU358861 | GU205370 | EU358901 | EU358941 |
| 33 | *Toxoptera odinae* | 050513HJ14 | EU358862 | GU205371 | EU358902 | EU358942 |
| 34 | *Schizaphis graminum* | 050804HJ2 | EU358858 | GU205372 | EU358898 | EU358938 |
